# Organized films

**DOI:** 10.3762/bjnano.7.35

**Published:** 2016-03-09

**Authors:** Maurizio Canepa, Helmuth Möhwald

**Affiliations:** 1 Department of Physics, University of Genova, via Dodecaneso 33, 16146 Genova, Italy,; 2 Max Planck Institute of Colloids and Interfaces, Am Mühlenberg 1, 14476 Potsdam, Germany

Langmuir–Blodgett (LB) films, prepared by transferring an amphiphilic molecular monolayer (Langmuir monolayer) from a water surface onto a solid substrate, represent both archetypal and prototypical “organized films” (OFs). Known since about 80 years, LB films can be considered from many points of view as one of the prominent predecessors of what we call nanoscience today [1]. Their impact on interface science over the last four decades was undoubtedly based on pioneering works such as the beautiful studies of Kuhn and Möbius on energy transfer between molecules arranged with angstrom precision [2], or the stimulating works of Roberts and Petty [3], which envisaged many applications. This was most notable in the fields of molecular electronics and biosensors, which were emerging in those times and are now undergoing flourishing development.

The interdisciplinary character of OFs, at the fortunately ill-defined borders between physical chemistry, chemical physics, biophysics and surface engineering, is a constant that marks the evolution of the field. In fact, one of us (H.M.) with his group entered the field many years ago, initially motivated by the biophysics of membranes and contributed to the development of new methods to characterize monolayers. Fluorescence microscopy and Brewster angle microscopy [4–7] were successfully employed to show that Langmuir monolayers exhibit an interesting domain structure on the micrometer scale with peculiar features due to the anisotropy of the interface. Surface X-ray diffraction revealed a multitude of ordered and disordered phases [8] that could be mapped on smectic liquid crystals.

These and other studies, while revealing the interesting two-dimensional physics of Langmuir monolayers, also brought to attention some important issues in obtaining films with high geometric order extending over micrometer length scales. On one hand, the domain structure causes grain boundaries, which are almost impossible to remove. Furthermore, the tight packing of alkane chains in classical LB films prohibits the incorporation of foreign molecules with high precision. This fact is a significant disadvantage if one aims to arrange a molecule in a controlled way for electronic and optoelectronic applications. In this respect, defined protein incorporation, for example, for designing a biosensor, seemed hopeless. Incidentally, these problems were largely shared by self-assembled monolayers (SAMs), as exemplified by the most popular examples of alkanethiols on Au or silanes and siloxanes on SiO_2_. These are yet another very broad class of OFs whose investigation proceeded in parallel with that of Langmuir films [9–10].

Clearly, the whole research sector benefited from the evolution of new analysis tools and surface spectroscopy methods, which allowed for characterization at all length scales. One may first think of monolayer-sensitive optical methods [11] or of the wealth of techniques based on third and fourth generation synchrotron sources. However, the most striking example is by far represented by the invention and extensive development of scanning probe methods in the late 1980s. With these methods, one could learn much more about details of the molecular structure and the surface order [12–13], penetrating the nanoscale realm. Furthermore, AFM in particular enabled continuous developments in manipulating and modifying films, opening the way to patterning and even to nanolithography methods [14]. This was a crucial step in the development of nanosensors and biochips, especially in new and emerging fields such as nanomedicine.

Coming back to LB films, irrespective of other technical difficulties in fabrication and long term stability, the problems mentioned above made such films less appealing for applications, emphasizing the need for exploration and invention of new processes of film fabrication. The most successful of these processes evolved during the habilitation of Gero Decher. In this process, popularly known as the layer-by-layer (LbL) technique, one consecutively adsorbs polyelectrolytes of opposite charge to form multilayers of nanometer thickness [15–16].

The studies were quickly extended to explore other types of (i) interactions (towards bio-specific ones and hydrogen bonding), (ii) adsorbents (from polymers to particles, proteins, polyvalent molecules), and (iii) substrates (from planar to colloidal particles and porous membranes). The know-how accumulated on OFs reinforced well-established links with bio-oriented research, as in the case of lipid–protein interactions [17–18] or in the development of functional capsules, opening interesting application perspectives [19–21]. Another side of the field of OF met the explosion of research on nano-objects such as nanoparticles, carbon nanotubes or the latest, graphene [22]. These have been used to build hybrid structures endowed with specific functionalities for applications in strategic technology fields as varied as theranostics, energy production and storage, and photovoltaics.

Similar trajectories were observed for SAMs, with increasing attention on new combinations of functional molecules and substrates, new analysis tools and new preparation methods [23], which varied significantly from the more traditional deposition from solution [24]. This is the case for MBE-like deposition in vacuum or UHV conditions [25], which is of current interest in the area of organic photovoltaics [26]. This also applies for biological molecules such as small amino acids, which allow fundamental and still open issues to be addressed about the subtle interplay between molecule–molecule and molecule–substrate interactions in determining the molecular networking at surfaces [27–29].

These few notes introduce this Thematic Series, which aims to present a partial overview of the current research on OFs. The articles emphasize the strong interdisciplinary character, the evident connections with nanoscience and nanotechnology, and some directions of expansion of the field of OFs.

This Thematic Series was conceived while organizing ECOF-14, the European Conference on Organized Films, which took place in Genova in July 2015, chaired by one of us (M.C.) and Ornella Cavalleri. This conference has an established tradition that dates back to a discussion meeting organized in 1986 by Helmuth Möhwald and Wolfgang Knoll in Munich and a follow-up meeting organized as a NATO workshop in Paris by André Barraud in 1988. These meetings finally evolved into a Europe-based conference, ECOF-3 (Mainz, 1990), organized by the Möhwald group. The number following the conference name abbreviation was preserved to indicate successive conferences, and since then, the conference has traveled all over Europe, with the next conference announced for Dresden, 2017. It still encompasses lively and fruitful discussions of about 200 participants, again this time with high scientific quality and openness to nearest neighbor fields, as the readers of this series are invited to verify.

Maurizio Canepa and Helmuth Möhwald

Genova and Potsdam, February 2016

## References

[1] Kuhn H, Möbius D (1971). Angew Chem, Int Ed Engl.

[2] Nakahara H, Fukuda K, Möbius D, Kuhn H (1986). J Phys Chem.

[3] Roberts G G, Petty M C, Baker S, Fowler M T, Thomas N J (1985). Thin Solid Films.

[4] Lösche M, Sackmann E, Möhwald H (1983). Ber Bunsen-Ges Phys Chem.

[5] Knobler C M (1990). Science.

[6] Hénon S, Meunier J (1991). Rev Sci Instrum.

[7] Hönig D, Möbius D (1991). J Phys Chem.

[8] Kjaer K, Als-Nielsen J, Helm C A, Laxhuber L A, Möhwald H (1987). Phys Rev Lett.

[9] Sabatani E, Rubinstein I, Maoz R, Sagiv J (1987). J Electroanal Chem Interfacial Electrochem.

[10] Laibinis P E, Whitesides G M, Allara D L, Tao Y T, Parikh A N, Nuzzo R G (1991). J Am Chem Soc.

[11] Canepa M, Bracco G, Holst B (2013). A Surface Scientist's View on Spectroscopic Ellipsometry. Surface Science Techniques.

[12] Schwartz D K, Garnaes J, Viswanathan R, Zasadzinski J A N (1992). Science.

[13] Poirier G E, Tarlov M J (1994). Langmuir.

[14] Xu S, Liu G-y (1997). Langmuir.

[15] Decher G, Hong J-D (1991). Makromol Chem, Macromol Symp.

[16] Lvov Y, Decher G, Möhwald H (1993). Langmuir.

[17] Maltseva E, Kerth A, Blume A, Möhwald H, Brezesinski G (2005). ChemBioChem.

[18] Canale C, Torrassa S, Rispoli P, Relini A, Rolandi R, Bucciantini M, Stefani M, Gliozzi A (2006). Biophys J.

[19] Caruso F, Caruso R A, Möhwald H (1998). Science.

[20] Diaspro A, Silvano D, Krol S, Cavalleri O, Gliozzi A (2002). Langmuir.

[21] Sukhorukov G, Fery A, Möhwald H (2005). Prog Polym Sci.

[22] Li X, Zhang G, Bai X, Sun X, Wang X, Wang E, Dai H (2008). Nat Nanotechnol.

[23] Love J C, Estroff L A, Kriebel J K, Nuzzo R G, Whitesides G M (2005). Chem Rev.

[24] Barth J V, Costantini G, Kern K (2005). Nature.

[25] Schreiber F, Eberhardt A, Leung T Y B, Schwartz P, Wetterer S M, Lavrich D J, Berman L, Fenter P, Eisenberger P, Scoles G (1998). Phys Rev B.

[26] Gottfried J M (2015). Surf Sci Rep.

[27] Kühnle A, Molina L M, Linderoth T R, Hammer B, Besenbacher F (2004). Phys Rev Lett.

[28] Cavalleri O, Gonella G, Terreni S, Vignolo M, Floreano L, Morgante A, Canepa M, Rolandi R (2004). Phys Chem Chem Phys.

[29] Gonella G, Terreni S, Cvetko D, Cossaro A, Mattera L, Cavalleri O, Rolandi R, Morgante A, Floreano L, Canepa M (2005). J Phys Chem B.

